# National Health Observatory: A tool to strengthen the health information system for evidence-based decision making and health policy formulation in Cameroon

**DOI:** 10.1016/j.hpopen.2022.100085

**Published:** 2022-12

**Authors:** Viviane Fossouo Ndoungue, Christie Tiwoda, Oumarou Gnigninanjouena, Serge Bataliack, Ebongué Mbondji, Aline Labat

**Affiliations:** a National Public Health Observatory, Yaoundé, Cameroon; b Ministry of Public Health, Yaoundé, Cameroon; c World Health Organization for the African Region, Brazzaville, Congo; d University of Pretoria, School of Health System and Public Health, South Africa; e Université libre de Bruxelles, School of Public Health, Belgium; f Health Systems Strengthening and Development (HSSD Group), Yaoundé, Cameroon

**Keywords:** National health observatories, Health policy formulation, Health information systems, Evidence-based decision making, Cameroon

## Abstract

Despite the consensus on the importance of evidence based health information for decision28 making and health policy formulation, quality information is not always available. This situation led the WHO in 2009 to recommend the establishment of an African Health Observatory and National Health Observatories. The WHO recommended 4 phases approach for the establishment of National Health Observatories was used in Cameroon: the preliminary phase, start-up, strengthening and full operation. Despite a non-optimal institutional positioning, the Cameroon’s Health Observatory is in the strengthening phase and contributes to evidence-based decision making in the health system. A strong political commitment is fundamental from the beginning of the project. The establishment of National Health Observatories calls for genuine national ownership, both of the process as a whole and of the tool itself. WHO should regularly assess the progress made in the establishment of National Health Observatories in different countries and provide feedback to them. A step-by-step approach, jointly validated by national stakeholders in a roll-out plan, would be more appropriate for the sustainability of the work of National Health Observatories.

## Highlights

• National Health Observatories are pillars of the health information system.
• The establishment of National Health Observatories calls for genuine national ownership, both of the process as a whole and of the tool itself.
• A step-by-step approach, jointly validated with national stakeholders in a roll-out plan, would be more appropriate for the sustainability of the work of the National Health Observatories.

## 1 Background

Knowledge transfer is a process that brings the knowledge producing and policy making communities closer together and focuses on improving the mechanisms for sharing, disseminating and exchanging knowledge between stakeholders through specific strategies [1]. The public health challenges facing health systems in the World Health Organization (WHO) African Region require effective health information management to ensure the effectiveness of the right to health and to improve the health status of populations. Optimal use of available technology in the field of health information can significantly increase the capacity of the public health system to make decisions in real time [2]. Timely and accurate data are needed to enable countries to assess the health status of populations, set priorities and monitor progress towards goals and targets, including the Sustainable Development Goals (SDG) and Universal Health Coverage (UHC) [3]. In Europe for example, the Observatory was able to demonstrate how the economic crisis has affected population health, presented evidence of the short- and long-term benefits of investing in disease prevention and health promotion, and showed how health impact assessment, coupled with effective governance, could influence decision making and support implementation, moving away from simple policy analysis [4].

Despite the consensus on the importance of knowledge transfer for decision-making, good quality information is not always available [5]. The gap between what health professionals know and what they should know to improve the health of the population remains considerable. Indeed, although the health information system is a fundamental pillar of health systems, the capacity to make the best use of health information to improve health is generally limited and unevenly distributed across the African Region [6]. Building such capacity is considered one of the first priorities in the primary health care approach to reforming national health systems and addressing the social determinants of health.

The recommendations contained in the Ouagadougou Declaration of 30 April 2008 on primary health care and health systems in Africa [7] and the Algiers Declaration of 26 June 2008 on research for health in the African Region [8] for the establishment of an African Health Observatory (AHO), later called in 2020 Integrated African Health Observatory (iAHO) and National Health Observatories (NHO), are in line with this. These Declarations committed signatory countries to develop and strengthen the use of evidence from their health systems to inform policy and decision making [8] through the establishment of an AHO and NHOs [7], [9], [10]. WHO started working on health observatories in the African Region in 2010 when it established the AHO. This followed the 2009 recommendation of the Regional Committee (AFR/RC59/5). In 2012, the Regional Committee (AFR/RC62/R5) requested WHO to assist Member States in establishing National Health Observatories (NHOs) [11], whose objective are to serve as a tool to track and monitor priority health issues in the region and to bring together key health actors for this purpose. It is one of the pillars of the framework for strengthening the health information system in the component “Promoting the use of data for decision-making”, in relation to the NHOs, to contribute to the collation and analysis, as well as the monitoring and evaluation of data at national level [6].

The WHO Regional Office for Africa provides support to countries to establish national NHOs based on the AHO prototype [11], [12], [13]. Thus, NHOs have been set up to perform four main functions: (i) gathering and storing data from different sources; (ii) analysing and monitoring health phenomena and trends, including progress towards internationally agreed goals such as the health MDGs; (iii) disseminating and sharing evidence and knowledge through analysis and synthesis of information; and (iv) supporting networking and community working to make better use of this data and knowledge in policy and decision-making [14]. Between 2011 and 2016, ten countries including Cameroon were selected by WHO, based on their location in the WHO Regional Office for Africa decentralized Inter-country Support Teams (ISTs) and their language of expression (English or French), to receive support to establish their NHO [13].

According to its organizational chart of 2013, the Cameroon Ministry of Health is responsible for the design and implementation of the Government's public health policy. The Ministry of Health is composed of a central administration, deconcentrated services, public health facilities and specialized technical committees and organizations. The NPHO is classified as a specialized technical body under the technical oversight of the Ministry of Health [15]. The Cameroon health system is composed of 10 regional health delegations, 190 health districts, 1802 health areas and about 5800 health facilities (51 % public and 49 % private) [16].

Cameroon, having adhered to the Ouagadougou and Algiers initiatives, has committed itself to the establishment of a NHO. This commitment was evidenced by the equipping and construction of a building inaugurated in November 2009 and the creation in 2010, by a decree of the Prime Minister, of the National Public Health Observatory (NPHO) [17]. This organisation, placed under the authority of the Minister of Health, has as its main mission, alert and health watch as well as the centralisation, analysis and implementation of social health information, databases and data banks relating to public health problems.

By 2020, NHOs have been fully implemented in seven of the ten countries that have been enrolled in this pilot process [3]. However, neither the process nor the functionality has been documented. Although a large number of models and theories for knowledge transfer interventions exist, the majority of them have not been tested, which means that their applicability and relevance are largely unknown [5]. In this perspective, sharing Cameroon's experience in setting up the NHO could be useful to other African countries in developing and formulating policies for setting up their Observatories. This experience sharing also follows the recommendations of the 62nd WHO Regional Committee for Africa which invited member states to undertake the monitoring of NHO and to record and share best practices [11].

## 2 Organisation and operation of the NPHO

Placed under the authority of the Minister of Health, the NPHO is a specialised technical body which comprises an executive body, the permanent secretariat and two advisory bodies, namely the Steering Committee and the Scientific Council.

The Steering Committee is the advisory and orientation structure of the NPHO, placed under the authority of the Minister of Health. It carries out the tasks entrusted to the NPHO and issues opinions on all matters submitted to it by the Minister of Health. It is also responsible for examining and adopting the annual workplan, the rules of procedure and the budget of the NPHO. It is composed of representatives of eight partner ministries, the National Institute of Statistics, WHO, the private faith-based health sector, the private secular health sector and civil society. This committee meets as needed when convened by its chairman. Since the creation of the NPHO, the Steering Committee has met three times for the political adoption of the NPHO's outputs.

The Scientific Council, headed by a chairman appointed by the Minister of Health, is an advisory body responsible for evaluating and validating the NPHO's scientific programs. It is made up of ten members appointed for a three-year term, renewable once, from the health, scientific research, higher education and development partner sectors. The Scientific Council meets at the request of the coordinator of the permanent secretariat of the NPHO. The council systematically carries out the scientific validation of the NPHO's products prior to their political adoption by the Steering Committee.

In order to carry out its tasks, the NPHO secretariat is organised into four sections: (i) a section for socio-health information, epidemiological surveillance and cooperation, (ii) a section for surveys, health and epidemiological studies, monitoring and forecasting, (iii) a general affairs section and (iv) an information technology section. The coordinator of the permanent secretariat is appointed by the Minister of Health.

## 3 Establishing the national public health Observatory (NPHO) in Cameroon

A guide to setting up NHOs was developed by WHO in 2016 [14] to provide guidance to countries wishing to develop their Observatory to strengthen their national health information system. This guide describes the objectives and functions of health observatories (collation, analysis and synthesis, sharing and networking), as well as their main components, and proposes a process for its implementation. It discusses the main issues to be addressed when implementing NHOs, such as organisational management, skill development and relationships with producers and users [14]. According to the guide, the implementation of NHOs takes place in four phases, the first three of which take one year each and are marked by a deliverable. These phases successively ensure (i) the establishment of the foundations on the basis of an analysis of the situation (preliminary phase) allowing for the development of an action plan, (ii) the strengthening of achievements (start-up phase) and (iii) the financing and consolidation of the observatory's functions (strengthening phase), set out in a strategic and operational plan. The last phase, that of full operation, marks the full expansion of the NHO [14]. The process of setting up the NPHO in Cameroon did not follow this linear path in terms of timeline and deliverable of each phase.

***The Preliminary Phase:*** In Cameroon, this phase lasted 1 year (2009–2010) as recommended in the guide for the establishment of NHOs. It began with the construction and equipping of the building to house the structure, inaugurated in 2009, as part of the African Development Bank health system development project. In 2010, the Cameroonian government marked its political commitment by creating and staffing the NPHO through a decree of the Prime Minister [17] which placed this structure under the technical oversight of the Minister of Health [15]. The technical staff of the NPHO (Permanent Secretariat), which has the status of a state civil servant, initially consisted of a coordinator specialised in public health, a computer analyst, and three public health specialists. Although it is not recommended in the guide to include support staff in this phase, this technical team was assisted by three administrative and financial staff and three support staff. Financial resources were also allocated for the operation of the NPHO. Although insufficient, they are largely charged to the budget of the Ministry of Health and supplemented by support from development partners. Due to these provisions, Cameroon was part of a cohort of ten pilot countries that benefited from the technical support of the WHO for the establishment of their national health observatory [13]. In this area, WHO worked in synergy in Cameroon with partners including the African Development Bank (ADB), the Bill & Melinda Gates Foundation, the German Technical Cooperation (GIZ) and the Center for Disease Control and Prevention (CDC) to strengthen the NPHO. This collaboration has made it possible to address the country's priorities for strengthening the health information system, and to recruit a local WHO technical advisor to provide technical support to the country [13]. The text creating the NPHO stipulates that its financing should also be ensured by the services it provides and that it can benefit from donations and legacies as well as various subsidies [17]; this has not been the case.

No situational analysis was conducted at this phase as recommended in the WHO guide to setting up NHOs. As a result, no action plan was developed to guide the establishment of the NPHO, which is a deliverable of the preliminary phase. This action plan was developed during the assessment of the overall situation of knowledge in the National Health Information System (NHIS), carried out 5 years after its creation, in 2015, during the national sensitisation workshop on the NPHO in the start-up phase. Similarly, the evaluation of the NHIS was not carried out at this stage to identify the strengths that the NPHO could rely on; identify the weaknesses of the system; and to set up a relevant and reliable collaboration model between the actors of the NHIS and the NPHO. This situation created conflicts of competence between the NPHO and certain technical departments of the Ministry of Health, such as the health information unit and the department in charge of epidemiological surveillance. The role of each structure was clarified during the national sensitisation workshop on the NPHO organised during the start-up phase.

***The start-up phase:*** It lasted six years (2011–2017), rather than one to two years as prescribed in the guide for the establishment of NHOs,as a result of the absence of a roadmap and began in 2011 with the strengthening of human resources. A multidisciplinary team consisting of a health economist, four public health specialists and a documentalist was gradually assigned to ensure the optimal functioning of the structure. The team was expanded to include: (i) public health specialists and epidemiologists with expertise in data analysis, both quantitative and qualitative, and the production of evidence-based data; (ii) health economics specialists; (iii) data managers with expertise in programming and website maintenance for the management of the digital platform; and (iv) a documentalist. The recruitment of these staff followed the civil service recruitment and assignment process. It was not done in a transparent manner and on the basis of the assessment of competences by independent experts as recommended in the guide [14]. Moreover, there is no regular performance-based assessment of state staff. In view of this situation, the NPHO has several times called on consultants or expertise from other technical directorates of the Ministry of Health to carry out certain activities. However, as part of the capacity building of countries through technical support from WHO, Cameroon has benefited from the training of its actors in observatory management tools.

**Fig. 1:**
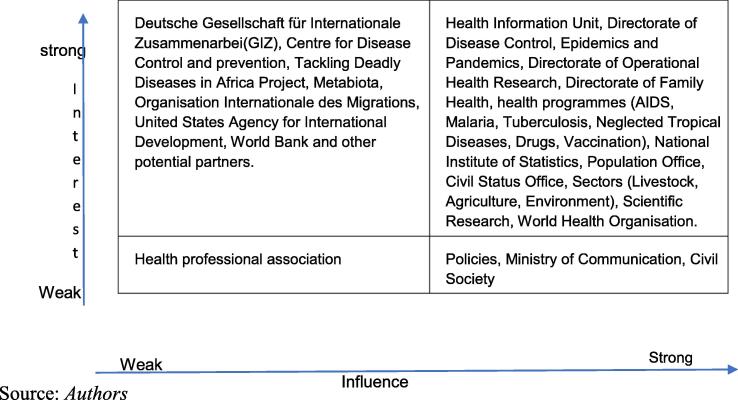
Matrix classifying NPHO stakeholders according to their interest and influence.

These include training in the use of Tableau software for data analysis, management and administration of the NPHO's digital platform, evaluation of the national health information system using the SCORE (Survey, Count, Optimize, Review, Enable) package, and training in the collection and analysis of health data, including data quality assessment using WHO tools. This extensive capacity building should enable the country to produce quality health information to feed into the NPHO's digital platform. In addition, a national workshop to raise awareness of the role and mission of the NPHO among NPHO stakeholders was held in 2015. This led to an analysis of the NPHO's stakeholders and the development of a five-year roadmap for strengthening the Observatory. Also, an evaluation of the NHIS aimed to: (a) identify the promising aspects of the system and the strengths on which the NPHO can rely; (b) identify the weaknesses of the system; (c) establish a relevant and reliable model of collaboration between the NHIS and the NPHO was also carried out during this workshop. This assessment focused on the Ministry of Health on the one hand, and the health sector in general on the other, to provide an overall picture of the knowledge needs and resources available in the country, which provided a basis for all the activities of the Observatory. Fig. 1 classifies the stakeholders in a matrix according to their level of influence and interest in the implementation of the activities of the NPHO of Cameroon.

The five-year roadmap for strengthening the NPHO, a deliverable of the preliminary phase, was drawn up during this phase. The initial outputs of the NPHO and the evaluation of the initial phase in preparation for the construction phase were not carried out in this phase as recommended by the guide due to the lack of follow-up to the roadmap. This lack of initial output from the NPHO has eroded the initial political commitment. In addition, the digital platform developed by the WHO, which was to host these outputs, had not yet been officially handed over to the country due to the cumbersome and slow administrative procedures. As these prerequisites were not met, the official launch of the NPHO, a deliverable of the start-up phase, was not delivered.

***The strengthening phase:*** Recommended to last for three to five years. Since 2017, the NPHO has been in its strengthening phase marked by initial productions using, among others, data published in the reports of demographic health surveys, household survey reports, national health accounts, multiple indicator cluster survey reports, health service delivery indicator survey reports and priority health program reports. In addition, the NPHO is always invited in highly technical platforms such as the weekly Integrated Disease Surveillance and Response for consultation and provision of technical guidance. The four main functions of the NPHO (collation, analysis and synthesis, sharing and networking) are being implemented as proposed in the guide. Now that all the functions are implemented in routine, the last phase, full operation, should start soon.

## 4 Achievements and challenges of the national public health Observatory in Cameroon

The legal framework of the NPHO has remained ambiguous (is it a program? is it a project? is it a technical directorate?) and its institutional positioning in the organisation chart of the Ministry of Health has not always facilitated its mission of gathering and centralising health information from other structures in the Ministry of Health and other government organisations, whose institutional positioning is superior. This ambiguity is accentuated by the absence in this text of equivalence of positions with those of the Cameroonian public service. This situation led to the demotivation of staff who found themselves administratively under-classified, and has made the NPHO unattractive to qualified human resources. The review of the NPHO's legal framework should be revised to remedy these shortcomings by giving this institution a legal personality and a financial and administrative autonomy, in particular the possibility of recruiting staff on the basis of their expertise as well as their scientific and technical skills. The NPHO in its current form has inherited a certain number of missions that were previously assigned to the National Epidemiology Committee (NEC). In fact, a national multi-sectoral and multi-disciplinary technical group was set up to carry out a situational analysis which should lead to the development of an action plan and proposals for the strategic choices of the structure to host the Observatory. As a result of this situational analysis, the NEC was chosen to host the NPHO. This committee's mandate were to advise on health matters; to identify health problems; to contribute to the organisation, promotion and financing of epidemiological studies in the country; and, on the basis of the results of the research, to make recommendations to the Minister of Health as well as to any other head of ministerial department or national body responsible for the development and implementation of health programs.

The NEC's areas of interest included disease control, epidemiological surveillance and health statistics. During the establishment of the NPHO, the missions of the NEC were merged with those of the NPHO, conferring to the observatory of Cameroon, additional missions to those recommended by WHO. Thus, the NPHO is the National Focal Point for the (IHR, 2005) in Cameroon, responsible for communication with the WHO for the purposes of the (IHR, 2005). In this capacity, it also ensures the promotion and vulgarisation of the (IHR, 2005), capacity building of national actors on the (IHR, 2005) as well as monitoring the implementation of the (IHR, 2005) in Cameroon. It is within this framework that the NPHO conducted the joint external evaluation of the capacities of the (IHR, 2005), the development of the national health security action plan, the mapping of resources allocated to health security at the national level and the annual evaluations of the implementation of the IHR in the country.

In order to fulfil its ***collation function***, the NPHO collects data relevant to public health and its determinants from the digital platforms of national and international actors in the NHIS and compiles them in a database/reports in the documentation services. However, the fragmentation of the NHIS by vertical programs that invest resources for short-term results does not allow the NPHO to sustain this aggregation function. The scaling up of the use of the District Health Information Software (DHIS2) as a single tool for routine data collection at the level of the NHIS and the establishment of the CHDC at the level of the NPHO have helped to strengthen this aspect. However, the sub-optimal functionality of DHIS2 (low completeness and promptness) does not allow the health indicators to be filled in with routine data at the Observatory level.

***The analysis and synthesis function*** of the NPHO has so far resulted in the compilation of national indicators in the “100 key health indicators monitoring report”, produced in 2017 [18] and 2019 [16]. A particular feature of the 2019 report is the integration of a focus on the SDGs and the UHC for a better visibility of trends and progress towards the achievement of the international and national targets set. The “Analytical Health Profile” [19] and a “Policy Brief on Measles Outbreaks in Cameroon” were also produced in 2017 to guide the decision maker.

***The sharing function*** provided by the NPHO has resulted in a contextualisation of the prototype digital platform developed by WHO. This platform thus serves as a one-stop shop for sharing health information and is accessible online at https://nho.minsante.cm/. It presents a clear picture of the country's health situation through the publication of thematic reports, dashboards, the country's health profile, policy briefs and other documents validated at the national level.

***The network function*** is ensured by the NPHO through the establishment in December 2016 of a national network of data producers, the “Cameroon Health Data Collaborative” (CHDC). The CHDC also constitutes a platform for sharing and exchanging health data. There was an initial high level of enthusiasm, with an average of 60 participants at monthly meetings and effective participation in activities, particularly for mapping data sources and funding in the SNIS, we note, however, that the CHDC has been operating intermittently over the past year due to a certain lack of motivation among members linked to the absence of strong leadership and activities that arouse the interest of data producers.

In order to ensure its mission of monitoring the implementation of the (IHR, 2005), Cameroon has defined a fifth function: health monitoring. In this respect, the NPHO led the joint external evaluation process of the (IHR, 2005) and the development of the National Health Security Action Plan (NAPHS) and ensured the establishment and strengthening of the national cross-border epidemiological surveillance system. The Observatory is also a member of the national epidemiological surveillance platform, where it is consulted on issues related to national health security. With the aim of consolidating its organisational and legal framework to better ensure its functions, the NPHO has undertaken the revision of its legal framework to establish it as an autonomous structure.

In order to guarantee the optimal functioning of health observatories, it is crucial to carry out a good analysis of all stakeholders and to continuously maintain their involvement according to their level of influence and interest in the Observatory. Box 1 summarises the involvement of the stakeholders of the NPHO.

Box 1 **Involvement of stakeholders in the NPHO of Cameroon, 2015.**

The objectives of high-influence but low-interest stakeholders, such as policy makers, are taken into account to ensure that they remain strong advocates. Aligning the objectives of the NPHO with the health sector strategy and health development plan helps to satisfy these stakeholders. In addition, a dashboard of key SDG and UHC indicators is available on the NPHO digital platform to inform policy makers.

Stakeholders with a strong influence and interest are closely involved in the implementation of the NPHO's activities. The Health Information Unit, the Directorate for Disease Control, Epidemics and Pandemics co-leads the Cameroon Health Data Collaborative (CHDC) network of data producers. Other stakeholders are invited to participate in exchanges on strategies for strengthening the NHIS within the CHDC and receive by e-mail the publications of the NPHO as well as other relevant publications on health information. Also, the annual reports of the national HIV, malaria and malaria control program; the reports of the demographic health surveys and household surveys; the national health accounts, the reports of the survey on health service delivery indicators; all produced by other NHIS actors are accessible through the NPHO's digital platform. The livestock, environment, agriculture, scientific research, higher education and civil society sectors are members of the NPHO's scientific council and steering committee. This approach helps to build strong relationships with the NPHO and ensure that their support is maintained. They are involved in decision-making and are regularly consulted.

Stakeholders with little influence and interest are involved from time to time in the activities of the NPHO to ensure that they remain at the forefront of their involvement as their interest and influence may change over time. The Cameroon Epidemiology Society (CaSE) receives all publications from the NPHO to encourage their interest, and through them, that of other professional bodies.

Stakeholders with little influence but strong interest are actual or potential sources of funding for the NPHO. GIZ, CDC, TDDA, Metabiota, IOM, USAID and the World Bank are regularly informed of activities through the NPHO's outputs. They are consulted on their areas of intervention and their contributions are used to improve the chances of success of the NPHO. The report of the biological and behavioural survey on HIV among key populations in Cameroon, published in 2016 by Metabiota, is published on the NPHO platform.

## 5 Conclusions

The establishment of the NHO requires significant political commitment. This commitment can be achieved through structured advocacy of the process, from the launch of the health observatory. The commitment of a minimum team is also crucial during the implementation to execute the project. The establishment of NHOs calls for genuine national ownership, both of the process as a whole and of the tool itself. The NPHO is an effective decision-making tool through its involvement in many of the Ministry of Health's decision-making bodies, and allows for the monitoring of trends and progress towards national and international goals through the annual publication of the 100 key health indicators report and the population health status profile. The creation and maintenance of a data production network is essential in order to have access to the different sources of information and to raise awareness of the need to align investments for an integrated NHIS. In Cameroon, the establishment of a network called the CHDC has helped to reduce the fragmentation of the national health information system through the sharing of information and the pooling of efforts for the implementation of certain activities (case of the Survey Delivery Indicators /Health Facility Assessment survey with the joint commitment of the Global Fund and the World Bank). The NPHO has a digital data and statistics platform, online publications on the health situation and trends in the country, and the country's analytical health profile is known. For optimal functioning, the institutional positioning and the legal and regulatory environment of the Observatory in the host institution must be clearly defined. It must also be free from any relationship or influence related to the administrative hierarchy with the producers and users of data within the NHIS. To attract and maintain the interest of decision-makers, NHO outputs must be early, relevant, regular and on subjects of national interest. Indeed, the first productions of the NPHO aroused a new interest in health information and demonstrated the importance of the NPHO. These first productions showed that decision-makers were thirsty for accurate information on public health. Their interest in NPHO publications was reflected in letters of congratulations from the very top and an increase in the NPHO's operating budget. WHO, as the Secretariat of the Member States in the region, should assist the country in coordinating the actions of international partners to ensure appropriate and sustainable mobilisation of resources, and maintain its support for the technical development of the observatory. Also, WHO should regularly assess the progress made in the establishment of NHOs in different countries and provide feedback to them. A step-by-step approach, jointly validated in a roll-out plan, would be more appropriate for the sustainability of the work of the NPHO.

## Funding

This research did not receive any specific grant from funding agencies in the public, commercial, or not-for-profit sectors.

## Declaration of Competing Interest

The authors declare that they have no known competing financial interests or personal relationships that could have appeared to influence the work reported in this paper.
